# Efficacy of the intranasal application of azaperone for sedation in weaned piglets

**DOI:** 10.17221/21/2023-VETMED

**Published:** 2023-04-26

**Authors:** Martin Svoboda, Jana Blahova, Jiri Jarkovsky, Adam Zacharda, Suzana Hajkova, Jonas Vanhara, Jan Vasek

**Affiliations:** ^1^Ruminant and Swine Clinic, Faculty of Veterinary Medicine, University of Veterinary Sciences Brno, Brno, Czech Republic; ^2^Department of Animal Protection and Welfare and Veterinary Public Health, Faculty of Veterinary Hygiene and Ecology, University of Veterinary Sciences Brno, Brno, Czech Republic; ^3^Institute of Biostatistics and Analyses, Faculty of Medicine, Masaryk University Brno, Brno, Czech Republic; ^4^Faculty of Veterinary Medicine, University of Veterinary Sciences Brno, Brno, Czech Republic

**Keywords:** behaviour, neuroleptic, pharmacodynamics, swine

## Abstract

The aim of the study was to compare the efficacy of the intranasal and parenteral administration of azaperone in order to achieve pig sedation. A total of 32 weaned piglets divided into 4 groups (8 piglets in each group) were used. Group A was injected intramuscularly (i.m.) with azaperone (Stresnil^®^, 40 mg/ml inj.; Elanco Animal Health) at a dose of 2 mg/kg of body weight (b.w.). Group B received a dose of 2 mg/kg b.w. of azaperone intranasally. Group C was given azaperone intranasally at a dose of 4 mg/kg b.w. Group D was given 1 ml of saline intranasally and served as the control group. The response to the defined stimulus (a blunt blow of a metal rod into a metal edge of a pen), the degree of salivation, movement level, body temperature and serum azaperone concentration were included in the trial. We found that in order to induce an adequate level of sedation comparable to the standard method of application, i.e., 2 mg/kg b.w. i.m., the intranasal administration of azaperone at a dose of 4 mg/kg body weight is required.

Pigs in intensive farming conditions encounter stressful situations that require the use of sedatives (Martinez-Miro et al. 2016). In common practice, pig sedation is standardly performed by the intramuscular (i.m.) injection of azaperone at a dose of 2 mg/kg of body weight (b.w.) (Porter and Slusser 1985). Azaperone is a butyrophenone neuroleptic, which exerts its effects mainly through antagonism toward the G-protein coupled dopamine D2 receptor which anti-adrenergic properties (Golan 2016). An interesting alternative possibility of administering the drug is the intranasal application of azaperone in piglets.

The advantage is the simplicity in the preparation of the application, its painlessness and efficiency. Thanks to this, the intranasal administration of drugs is being increasingly used in human medicine, especially in children (Vranikova and Gajdziok 2015). The intranasal application makes it possible to achieve a faster and higher level of absorption of the drug due to the high permeability, large blood supply, low enzymatic activity of the environment and by the fact that the absorbed substances avoid the so-called first pass metabolism in the liver when the concentration of the drug is reduced even before it enters the systemic circulation (Xu et al. 2020). In addition, the olfactory area of the nasal cavity, due to its proximity to the brain, enables the absorption of the drug directly into the brain and cerebrospinal fluid via the olfactory neurons. This bypasses the blood-brain barrier (Graff and Pollack 2005).

A similar benefit of this form of application can also be assumed in improving the welfare of piglets. The effort is to verify other options that can be used to improve the welfare conditions of pig farming.

In the available literature, there are, so far, no comparative data available on the clinical efficacy and optimal dosing of intranasal azaperone in pigs.

Therefore, the aim of this paper was to compare the efficacy of the intranasal and intramuscular administration of azaperone in achieving sedation in piglets.

## MATERIAL AND METHODS

The study was approved by the Ethics Committee of the University of Veterinary Sciences Brno. The study was a prospective randomised and double blinded one. The experiment was carried out on a school farm in Nový Jičín (Czech Republic). A total of 32 piglets were used after weaning, i.e., at the age of approximately 28 days. Only gilts were used in the study. Before the start of the experiment, the piglets were marked with a coloured ear tag in the right earlobe and the experimental animals were randomly divided into 4 groups of 8 piglets each. The randomisation was achieved by drawing the animals using numbered papers.

Group A (body weight, mean ± standard deviation, 8.80 ± 0.91 kg) was administered azaperone (Stresnil^®^, 40 mg/ml inj.; Elanco Animal Health, Greenfield, USA) i.m. at a dose of 2 mg/kg b.w. (standard procedure in practice). Group B (8.20 ± 0.65 kg was given azaperone (Stresnil^®^, 40 mg/ml inj.; Elanco Animal Health, Greenfield, USA) intranasally at a dose of 2 mg/kg of body weight. Group C (8.10 ± 0.92 kg) was given azaperone (Stresnil^®^, 40 mg/ml inj.; Elanco Animal Health, Greenfield, USA) intranasally at a dose of 4 mg/kg of body weight. Group D (control, 8.90 ± 0.98 kg) was given intranasally 1 ml of physiological solution. For the intranasal application, an LMA MAD Nasal intranasal drug applicator (Bexamed s.r.o., Prague, Czech Republic) was used.

Blood was taken from the piglets before the application of azaperone (0) and at intervals of 30, 90 and 240 min after the application. The blood samples were taken from the *vena cava cranialis* and the serum was obtained. The concentrations of azaperone were determined in the blood serum using the enzyme-linked immunosorbent assay (ELISA) method with a commercial kit produced by EuroProxima (Arnhem, the Netherlands).

The clinical effectiveness of the different forms of the azaperone application to achieve sedation in piglets was evaluated in all the experimental groups based on the response to loud stimulation by a blunt blow of a metal rod on a metal edge of the pen.

This indicator was evaluated every 15 min during the first hour after application (it was necessary to capture the onset and maximum level of sedation), then at 30-min intervals until the end of the experiment (240 min) in all the piglets in the following stages: 0 – high degree reaction (jump, escape), 1 – medium degree reaction (no hops, but reaction – step aside, head movement, muscle tremors, raising ears), 2 – no reaction.

Furthermore, the physiological functions of the pigs were assessed (0, 30, 90, 180 and 240 min after the application), which included the movement, degree of salivation and body temperature.

The degree of movement was evaluated as follows: 0 – normal movement, 1 – ataxic or less active, 2 – lying down.

The degree of salivation was evaluated as follows: 0 – no salivation, 1 – moderate level of salivation (discharge of a small amount of saliva from the corners of the mouth), 2 – high level of salivation (an overflow of saliva from the mouth, drooling).

The piglets’ body temperature was measured with a digital thermometer inserted into the rectum, which was recorded in degrees Celsius (°C).

The piglets were fed standard pelleted feed mixtures. Due to the use of a non-injectable form of administration for the preparation containing azaperone, and the higher than the recommended doses, i.e., off-label use, the pigs were monitored from the point of view of the withdrawal period for at least 56 days after the end of the experiment and then sent to the slaughterhouse.

### Statistical analysis

The Kruskal-Wallis test was used to analyse the statistical significance of the differences among the groups. It was followed by the Mann-Whitney test for the between the groups comparison. The Friedman test followed by the Wilcoxon test was applied for the analysis of the statistical significance of the differences among and between time points. The analysis was performed using SPSS v28.0.1.1 (IBM, USA).

## RESULTS

The results are presented as the mean ± standard deviation (SD) in the case of the serum concentration of azaperone and the rectal temperature. In case of the assessment of the sedation, movement and salivation, the results are presented as the percentage of the piglets in the group belonging to a certain grade.

### Evaluation of the sedation level – response to the loud stimulation

The results of the evaluation of the sedation level are presented in Table 1.

**Table 1:** Response to the loud stimulation

Observation time (min)	Groups														
	A (2 mg/kg b.w. i.m., *n* = 8; %)				B (2 mg/kg b.w., *n* = 8; %)				C (4 mg/kg b.w., *n *= 8; %)				D (control, *n* = 8; %)		
	0	1	2		0	1	2		0	1	2		0	1	2
15	0	50	50		62.50	37.50	0		37.50	62.50	0		100	0	0
30	0	37.50	62.50		50	50	0		37.50	62.50	0		100	0	0
45	0	25	75		12.50	25	62.50		12.50	25	62.50		87.50	12.50	0
60	25	50	25		0	50	50		0	37.50	62.50		75	25	0
90	37.50	50	12.50		25	50	25		0	25	75		50	37.50	12.50
120	62.50	37.50	0		75	25	0		0	62.50	37.50		62.50	37.50	0
150	75	25	0		62.50	37.50	0		25	62.50	12.50		50	50	0
180	87.50	12.50	0		87.50	12.50	0		62.50	37.50	0		50	37.50	12.50
210	62.50	37.50	0		75	25	0		75	25	0		25	62.50	12.50
240	25	75	0		75	25	0		75	25	0		12.50	62.50	25

The results are presented as the percentage of piglets in the group belonging to a certain grade

0 – high grade reaction (e.g., jumping, running); 1 – medium grade reaction (e.g., moving the head); 2 – no response

In the case of the intramuscularly applied azaperone (group A) at a dose of 2 mg/kg, the onset of the sedation occurred in all the piglets in the group as early as 15 min after the application (a high-grade reaction was not detected in any piglet) and the induced sedation lasted until the 45^th^ min from the start of the experiment. In the 60^th^ min, 25% of the piglets showed a high-grade reaction.

In the groups with the intranasal application (groups B and C), a decreasing tendency in the intensity of the response to a loud stimulation was found with the increasing applied dose of azaperone.

In the case of the intranasally applied azaperone at a dose of 2 mg/kg, the onset of the sedation occurred in the entire group of piglets 60 min after the application. In the 90^th^ min, 25% of the piglets showed a high-grade reaction.

With the intranasally applied azaperone at a dose of 4 mg/kg, the onset of the sedation occurred in all the piglets in the group 60 min after the application and lasted in the whole group until the 120^th^ min from the start of the experiment. At 150 min, 25% of the piglets showed a high-grade reaction.

### Physiological parameters

Furthermore, the physiological functions of the piglets were assessed, which included the degree of salivation, movement, and body temperature. The results are given in Table 2.

**Table 2:** Results of the vital parameters

Groups	OT (min)	Movement level (%)			Body temperature (°C)
		0	1	2	
A (*n* = 8) 2 mg/kg b.w. i.m.	0	100	0	0	38.7 ± 0.3
	30	0	100	0	38.8 ± 0.2
	90	0	25	75	38.5 ± 0.5
	180	50	50	0	39.0 ± 0.3
	240	100	0	0	38.9 ± 0.3

B (*n* = 8) 2 mg/kg b.w.	0	100	0	0	38.8 ± 0.9
	30	25	75	0	38.6 ± 0.5
	90	0	87.50	12.50	38.6 ± 0.7
	180	75	25	0	39.0 ± 0.2
	240	100	0	0	39.0 ± 0.4

C (*n* = 8) 4 mg/kg b.w.	0	100	0	0	39.1 ± 0.2
	30	0	62.50	37.50	38.8 ± 0.3
	90	0	25	75	38.5 ± 0.6
	180	12.50	75	12.50	39.1 ± 0.3
	240	100	0	0	39.0 ± 0.4

D (*n* = 8) control	0	100	0	0	39.0 ± 0.2
	30	100	0	0	39.0 ± 0.5
	90	100	0	0	39.3 ± 0.4
	180	100	0	0	39.0 ± 0.4
	240	100	0	0	38.9 ± 0.3

The results are presented as the percentage of the piglets in the group belonging to a certain grade. Movement score: 0 – normal movement; 1 – ataxic or less active; 2 – lying down

Body temperatures are presented as the mean plus standard deviation

OT = observation time

### Degree of salivation

In all the experimental groups, the level of salivation remained at the same level (0 – no salivation) during the entire trial.

### Movement level

In the case of the intramuscularly applied azaperone (group A) at a dose of 2 mg/kg, ataxic or less active piglets were found from the 30^th^ to the 180^th^ min. The piglets were detected to be lying down only in the 90^th^ minute. From the 180^th^ min, no piglets were observed to be lying down.

With the intranasally applied azaperone at a dose of 2 mg/kg, ataxic or less active piglets were also detected from the 30^th^ to the 180^th^ minute. Lying The piglets were also detected to be lying down only in the 90^th^ minute. From the 180^th^ min, no piglets were observed to be lying down.

In the group of the intranasally applied azaperone at a dose of 4 mg/kg, ataxic or less active piglets were also found from the 30^th^ to the 180^th^ minute. The piglets were detected to be lying down from the 30^th^ to the 180^th^ minute. From the 240^th^ min, no piglets were observed to be lying down.

### Body temperature

During the experiment, we also measured the body temperature. The body temperatures in all the groups were within the physiological range in all the periods of the experiment. Within the individual groups, there were no statistically significant fluctuations during the experiment.

### Serum concentrations of azaperone

The results of the concentration of azaperone in the serum are presented in Table 3.

**Table 3:** Concentration of azaperone in the blood serum (ng/ml)

Blood collection time (min)	Groups			
	A (2 mg/kg b.w. i.m., *n* = 8)	B (2 mg/kg b.w., *n* = 8)	C (4 mg/kg b.w., *n* = 8)	D (control, *n* = 8)
0	n.d.^x^	n.d.^x^	n.d.^x^	n.d.^x^
30	156.5 ± 61.5^x^	77.74 ± 27.1^y^	128.4 ± 52.5^x^	n.d.^x^
90	75.1 ± 20.9^x^	35.5 ± 14.8^y^	71.3 ± 40.5^x^	n.d.^x^
240	28.1 ± 6.6^x^	15.8 ± 5.2^y^	28.2 ± 8.3^x^	n.d.^x^

Data are expressed as the mean ± standard deviation

Means with the same blood collection time and row lacking a common superscript letter (x, y, z) differ significantly (*P* < 0.05)

n.d. = non-detected – azaperone concentration below the detection limit – 0.785 ng/ml

As expected, no azaperone was detected in the control group. The maximum concentrations of azaperone in the blood serum were reached in all the groups 30 min after the application (time to maximum serum concentration, T_max_). The maximum serum concentrations (C_max_) in groups A, B and C reached the values of 156.5 ± 61.5, 77.74 ± 27.1, 128.4 ± 52.5 ng/ml, respectively. From the 30^th^ to the 240^th^ min, there was a significant decrease in the concentration of azaperone in the blood serum in all the experimental groups of piglets. In all the periods of the experiment, the azaperone concentrations in groups A and C were significantly higher than in group B. No statistically significant differences in the azaperone concentrations between groups A and C were found during the trial.

## DISCUSSION

It is well known that increased stress situations may have a negative impact on the weight gain of pigs as well as on other production parameters (feed conversion, the number of weaned piglets, piglet mortality, etc.) (Gonyou et al. 1988). Stress in breeding sows can negatively affect reproduction (Etim et al. 2013).

Stress can also affect the boar’s reproductive functions (Kamanova et al. 2021). Piglets are especially sensitive to stressors at the early weaning stage. This includes separation from their dam and changes in their feed from liquid to solid (Sureshkumar et al. 2022). The weaned piglets’ stress can be a significant predisposition to diarrhoea (Lin et al. 2022).

Swine stress can also impair their immunity and cause increased susceptibility to diseases (Kelly 1985; Martinez-Miro et al. 2016). Other problems which may occur under conditions of intensive pig farming are behavioural disorders. These include cannibalism, fighting for rank in the social hierarchy and the puerperal neurosis of sows (Ruediger and Schulze 2012; Martinez-Miro et al. 2016). The negative effects of aggression on the productivity found in pigs (McGlone and Curtis 1985) can be explained by direct effects, such as energy expenditure or costs of recovery from injuries, and due to the resulting social order (Gonyou et al. 1988). The use of sedatives can reduce the negative effects of these factors (Dantzer 1977).

The intramuscular azaperone administration shows a short duration of action of the active substance. It was found by Jones (1972) that the intramuscular azaperone administration achieves the maximum efficiency after 15 min in young pigs, after 30 min in adult pigs and the duration of action is from 2 h to 4 hours. In the case of our study, we obtained similar results after the intramuscular administration of azaperone. We found that in order to induce an adequate level of sedation comparable to the standard i.m. administration (2 mg/kg b.w.), the intranasal administration of azaperone at a dose of 4 mg/kg body weight is required. This finding corresponds with the data we found when measuring the concentration of azaperone in the blood serum, i.e., the concentrations of azaperone in the blood serum in groups A and C were statistically comparable in all the periods of the trial.

According to the European Agency for the Eva-luation of Medicinal Products (EMEA 1997), azaperone reaches the maximum plasma concentrations within 30 min after the intramuscular administration in pigs at a dose of 1 mg/kg b.w. In a study conducted in rats, Heykants et al. (1971) found that the maximum blood concentrations of azaperone were reached 30 min after the i.m. administration which then decline rapidly over the following 4 hours. In the case of the intranasal and intramuscular azaperone administration in our experiment, the results were comparable, i.e., the maximum blood concentrations of azaperone were detected 30 min after administration (T_max_) and then declined rapidly until the end of the trial.

Other physiological parameters were included in the study (degree of salivation, motoric activity, rectal temperature). In the available literature, there is no data about the influence of the intranasal administration of azaperone on these parameters.

Nishimura et al. (1993) found increased salivation after azaperone administration (i.m.) at a dose of 8 mg/kg b.w.). In our experiment, no change in salivation was observed either after the intramuscular or intranasal azaperone administration.

Holzchuh and Cremonesi (1991) found that the muscle tone can be diminished after azaperone administration. In our experiment, we obtained similar results. In the case of the intramuscularly applied azaperone, the piglets were detected to be lying down in the 90^th^ minute. With the intranasally applied azaperone at a dose of 2 mg/kg, the piglets were also detected to be lying down only from the 90^th^ minute. In the group of intranasally applied azaperone at a dose of 4 mg/kg, the piglets were detected to be lying down from the 30^th^ to the 180^th^ minute.

It was found that the rectal temperature decreased by 1–2 °C during the 4 h following the intramuscular azaperone administration after the parenteral administration at a dose of 5 mg/kg b.w. (Marsboom and Symoens 1968). No statistically significant changes in the rectal temperatures were found either after the intramuscular or intranasal administration in our study.

Intranasal sedation in pigs has, so far, only been described for midazolam. A study by Lacoste et al. (2000) demonstrated that intranasally administered midazolam at a dose of 0.2 mg/kg was effective in inducing sedation and anxiolysis in piglets. No data are currently available in the available literature regarding the use of intranasal azaperone for sedation in pigs. There are only two studies where azaperone was used as part of an anaesthetic mixture for the intranasal application to anaesthetise suckling piglets during castration (Axiak et al. 2007; Becker et al. 2021). However, in both mentioned studies, the absorption of azaperone was not separately evaluated. The intranasal application of pharmaceuticals is used both in human and veterinary medicine. The advantage is that irritations or painful injections can be omitted.

According to human studies (Holsti et al. 2007; Wolfe and Braude 2010), the intranasal administration is not more difficult than administration through injection, while accidental injuries with needles are excluded.

An important feature of the intranasal administration is the high permeability and the close proximity to the central nervous system (CNS). The mucosa of the nose provides a huge resorptive surface and greater permeability (Miller et al. 2008; Kerr et al. 2009). In addition, the drugs can also reach the brain directly via the olfactory or trigeminal nerve and, thus, the blood-brain barrier can be circumvented with the intranasal application (Hanson and Frey 2008).

Therefore, the quick onset of the effects, comparable to parenteral injections, is to be expected after intranasal administration.

Contradictory to this assumption, we obtained different results in our experiment. This discrepancy can be explained as follows: The ability to absorb by the intranasal route can be affected by a number of factors. The absorption capacity from the nasal cavity is limited so that the solubility and the potency of the drug can be the limiting factor. The clearance inside the nasal cavity is very rapid. Moreover, the extent and rate of the drug absorption in nasal drug delivery can be influenced by its metabolic stability, permeation mechanisms, and formulation of the drug (Erdo et al. 2018).

Our results show that intranasally administered azaperone is absorbed and causes sedation in piglets. It is obvious, from our results, that by increasing the dose of the intranasally administered azaperone, the duration of the sedation is longer. It can be concluded that, to induce an adequate level of sedation comparable to the standard method of application, i.e., 2 mg/kg b.w. i.m., the intranasal administration of azaperone at a dose of 4 mg/kg body weight is required.
